# An Approach to Neurometabolic Epilepsy in Children with an Underlying Neurometabolic Disorder

**Published:** 2020

**Authors:** Parvaneh KARIMZADEH, Parinaz HABIBI

**Affiliations:** 1 Department of Pediatric Neurology, Pediatric Neurology Research Center, Research Institute for Children’s Health, Shahid Beheshti University of Medical Sciences, Tehran, Iran.; 2 Pediatric Neurology Department, Mofid Children’s Hospital, Faculty of Medicine, Shahid Beheshti University of Medical Sciences, Tehran, Iran; 3 Pediatric Neurology Department, Tabriz Children Hospital, Tabriz University of Medical Sciences, Tabriz, Iran.

**Keywords:** Inborn errors of metabolism, Neurometabolic disorders, Epilepsy

## Abstract

**Objective:**

Inborn errors of metabolism (IEM) are rare conditions, with an overall incidence of 1 per 1000 births. Approximately 40-60% of IEM cases present with epilepsy as one of the main clinical presentations of the disease. A substantial number of these patients require timely and accurate diagnosis, besides specific treatment to prevent the irreversible outcomes.

**Materials & Methods:**

In this two-year retrospective study, a total of 128 patients with documented neurometabolic disorders were selected and evaluated in Mofid Children Hospital of Tehran, Iran, using a questionnaire to investigate the prevalence of epilepsy and seizure phenotypes. The collected data were evaluated in SPSS version 23.

**Results:**

Seizure was reported in 49% (63/128) of the patients. A single episode of seizure occurred in 7 (7%) patients. The prevalence of epilepsy was estimated at 42% (54/128). The most common seizure types were generalized tonic-clonic (43%), tonic (22%), and myoclonic (10%), respectively. Epilepsy was refractory in 30% (16/54) of the patients, and the mean number of administered anti-seizure drugs for refractory cases was 3.2. Overall, 50% of refractory cases had mixed-type seizures, and 25% had generalized tonic-clonic and myoclonic seizures.

**Conclusion:**

Neurometabolic disorders are rare, but treatable causes of epilepsy. A considerable number of patients (42%) in the current study presented with epilepsy as a clinical feature of IEM.

## Introduction

Neurometabolic disorders comprise a heterogeneous group of genetic defects, which interrupt biochemical pathways via three main mechanisms: 1) enzymatic dysfunction; 2) disturbance of cellular transmembrane transport; and 3) mitochondrial dysfunction. Inborn errors of metabolism (IEM) are rare conditions, with an overall incidence of 1 per 1000 births. They present with diverse clinical phenotypes, but often have neurological presentations. Epilepsy is a more common presentation of IEM, which occurs in approximately 40-60% of IEM cases as one of the main neurological signs **(**1**-**12**).**

In a previous study, which aimed to elucidate the genetic architecture of IEM involving epilepsy or seizure, a total of 880 human genes were identified. It was found that 373 (42%) genes were associated with IEM, and a substantial number (26%; 97/373) had specific treatments (13**)**. In addition, various clinical patterns may be associated with IEM, ranging from occasional seizures triggered by epilepsy to epileptic encephalopathy. Seizure type may be a valuable clue for determining the underlying etiology **(**14**-**17**)**

## Materials & Methods

This descriptive cross-sectional study was performed on patients with a documented diagnosis of neurometabolic disorders from September 2017 to May 2019. Patients were selected and evaluated in Mofid Children Hospital, Tehran, Iran. Diagnosis was confirmed according to the patient’s clinical history and confirmed by genetic studies in the majority of cases. 

The required information for this study was extracted from the patients’ archived documents, using a prepared questionnaire. The questionnaire included demographic data, pre/perinatal history, family history and consanguinity, clinical presentations of the disease, seizure characteristics, paraclinical findings, treatment response, and disease course; other supplementary data were also documented. Paraclinical evaluation was performed based on the speculated diagnosis. The basic diagnostic tests included brain magnetic resonance imaging (MRI), brain magnetic resonance spectroscopy (MRS), metabolic screening tests, and urine organic acid test, while the specific diagnostic tests included enzymatic assay or specific investigations. 

Definite diagnosis was confirmed via direct sequencing in the majority of cases and whole exome sequencing in others. Data were analyzed in SPSS version 23. Different diseases were identified in the patients (31 disease types). Consequently, for the ease of study and investigation, they were categorized into six main groups according to the Society for the Study of IEM (SSIEM 2012) guidelines: 1) amino acid metabolism defects; 2) lysosomal disorders; 3) peroxisomal disorders; 4) mitochondrial disorders; 5) fatty acid metabolism defects; and 6) transmembrane transport defects.

## Results

A total of 128 patients with a documented diagnosis of neurometabolic disorders were enrolled in this study. Based on the results, 59% of the subjects were male, and 41% were female. The mean age of subjects in the first visit was 4.94±0.54 years. Also, the mean age of initial symptom presentation was 2.55±0.44 years. The final diagnosis was established at the age of 5.1±0.53 years. Overall, 74% of the patients were children of consanguineous parents. Similar disease presentations were reported in 30% of patients with a family history of the disease. Direct gene sequencing was the leading method of definite diagnosis in 71% of cases, while whole exome sequencing was performed in 22% of cases. Also, clinical findings, neuroimaging, and metabolic screening were used for the definite diagnosis of 7% of cases. 

The prevalence of each disease category was as follows: amino acid metabolism defects, 12%; lysosomal disorders, 64%; peroxisomal disorders, 9%; mitochondrial disorders, 14%; fatty acid metabolism defects (one patient with carnitine deficiency), 0.5%; and transmembrane transport defect (one patient with Menkes disease), 0.5% (Tables 1-4).

Seizure was reported in 49% (63/128) of the patients. A single episode of seizure was reported in 7 (7%) patients. Seizure was triggered by fever in five patients, by gastroenteritis without fever in one patient (glutaric aciduria type I), and unprovoked in two patients (L2-hydroxyglutaric aciduria and pyruvate dehydrogenase deficiency) (Table 5).

 The prevalence of epilepsy was 42% (54/128) among patients. According to the history of seizure type, focal onset was reported in 25% of the patients. Seizure was generalized in 47% of cases. However, the type of seizure onset was not determined because of remote occurrence, recall bias, and lack of medical documentation. The most common seizure types were generalized tonic-clonic (GTC) (43%), tonic (22%), and myoclonic (10%), respectively. 

The initial electroencephalography (EEG) was inaccessible in 30% of the patients. There was no abnormal finding in 40% of cases, while 60% had abnormal, but not characteristic findings (e.g., spike-and-wave discharges and random or occasional epileptic discharges). Epilepsy was refractory in 30% (16/54) of cases, and the mean number of administered anti-seizure drugs for refractory cases was 3.2. Overall, 50% of refractory cases had mixed-type seizures, while 25% had GTC and myoclonic seizures; other seizure types constituted the minority. The characteristics of epilepsy in each group are presented separately in Table 6. The most commonly administered anti-seizure drugs were primidone, phenobarbital, clobazam, and levetiracetam, respectively.

**Table 1:** Diseases reported in group 1

**Disease**	**Number of patients**
Glutaric aciduria type 1	2
Isovaleric acidemia	1
Propionic acidemia	1
Asparaginase synthetase deficiency	1
Biotinidase deficiency	1
Canavan disease	5
Sulfite oxidase deficiency	1
L2-hydroxyglutaric aciduria	3
Total	15

**Table 2:** Diseases reported in group 2

**Disease**	**Number of patients**
Metachromatic leukodystrophy	6
Krabbe disease	2
Niemann-Pick disease type C	59
Late-onset GM1 gangliosidosis	1
Juvenile GM1 gangliosidosis	3
Tay-Sachs disease	3
Sandhoff disease	3
Late-onset GM2 gangliosidosis	4
Sanfilippo syndrome	1
Total	82

**Table 3:** Characteristics of patients in group 3

**Drugs**	**Seizure type**	**Seizure**	**Initial findings**					**Mean age of disease onset (years)**	**Number of patients**	**Adrenoleukodystrophy type**
			Addison’s disease	Seizure	Gait problem	Visual problem	Behavioral problem			
Phenobarbital	GTC	1	-	1	2	1	2	4.5	6	Childhood
Valproic acid, topiramate, and phenobarbital	GTC	2	1	1	1	1	-	13	4	Juvenile
Valproic acid	GTC									

**Table 4:** Patients categorized in group 4

**Number of patients **	**Disease**
4	Mitochondrial complex 1 defect
3	Mitochondrial complex 2 defect
1	Mitochondrial complex 3 defect
1	Mitochondrial complex 4 defect
1	mtDNA deletion syndrome
1	Kearns-Sayre syndrome
1	Coenzyme Q10 deficiency
1	MEGDEL syndrome
1	Multiple mitochondrial dysfunction
2	Pyruvate dehydrogenase
1	Pyruvate carboxylase

**Table 5:** Seizures triggered by fever

**Disease**	**Age of seizure occurrence**	**Seizure progress**	**Medical response (ASD number)**	**Other findings**
Asparagine synthetase deficiency	6 months	Refractory	5	Fever after vaccination
Metachromatic leukodystrophy	3.5 years	Single episode	-	-
Juvenile GM1 gangliosidosis	2.5 years	Single episode	-	Regression
Juvenile GM2 gangliosidosis	11 years	Single episode	-	Regression
Childhood adrenoleukodystrophy	5 years	Single episode	-	Adrenoleukodystrophy symptoms after two years
Pyruvate dehydrogenase deficiency	18 months	Refractory	3	Ataxia and metabolic crisis
Kearns-Sayre syndrome	18 months	Single episode	-	Ptosis, diabetes, and sensory-neural hearing loss

**Table 6:** Epilepsy characteristics in the groups

	**Epilepsy prevalence**	**Refractory epilepsy**	**Number of refractory cases**
Group 1	7/15 (46%)	2/7 (28%)	Asparagine synthetase deficiency Biotinidase deficiency
Group 2	43/82 (52%)	10/43 (23%)	Niemann-Pick disease type C (8) Metachromatic leukodystrophy (1) Juvenile GM2 gangliosidosis
Group 3	3/11 (27%)	1/3 (33%)	Adrenoleukodystrophy
Group	3/18 (16%)	3/3 (100%)	MEGDEL syndrome Pyruvate dehydrogenase Pyruvate carboxylase

## Discussion

Although IEM is a rare cause of epilepsy, seizure and epilepsy are common in IEM (40-60%). The prevalence of seizure and epilepsy in the current study was 42%, which is compatible with previous studies. Management of epilepsy in IEM cannot be accomplished unless the underlying etiology is recognized and treated. Pyridoxine-dependent epilepsy and biotinidase deficiency are some paramount examples **(**9**) **.Many of these disorders are amenable to specific treatment (97/373), and timely and accurate diagnosis is necessary to prevent the irreversible outcomes. In the present study, refractory epilepsy was controlled via biotin administration in patients with biotinidase deficiency (20 mg/day), and the administered anti-seizure drugs were successfully tapered off (four drugs tapered to two). The EEG findings did not indicate any clinical resolution, and the patients were responsive to pulse methylprednisolone administration (30 mg/kg/d for five days).

A wide range of clinical and EEG patterns of epilepsy may be suitable clues for IEM diagnosis, including burst suppression pattern, hypsarrhythmia, modified hypsarrhythmia associated with myoclonus, and paroxysmal responses to photic stimulation at low frequencies. However, such findings were not evaluated in our study due to several reasons. First, our hospital is a tertiary referral center, where most patients are not visited immediately after the disease onset, and we usually miss some initial critical data. Second, poor or unqualified documentation in some cases might be influential in the results. Third, the majority of patients in our study had Niemann-Pick disease type C (NPC). Since there was possible access to the filter paper method of screening in other centers and our center is a referral center for lysosomal diseases, there is a possibility of selection bias. Finally, this was a retrospective study. Therefore, to obtain all the required data, a prospective study must be designed.

Seizures may present with numerous clinical features. They may be initial and cardinal signs, occasional events triggered by fever or acute illness, or even a single event with no recurrence. In the current study, seizure provoked by fever was the initial presentation in seven patients. Although it is not common, in the context of febrile seizures, it is necessary to check the patient’s full medical background, family history, and physical examinations to explore possible metabolic etiologies (18-20). 

Reports of refractory epilepsy in the literature are contradictory due to different definitions, different etiologies, and different settings. However, the overall prevalence ranges from 20% to 40%. Attention must be paid to the patient’s partial or poor response to anti-seizure drugs in the presence of an underlying metabolic etiology. The prevalence of refractory epilepsy was 30% in the present study according to the International League against Epilepsy (ILAE) 2017 definition of refractory epilepsy, which is not higher than the prevalence reported in previous studies. There was a considerable difference between the prevalence of epilepsy (16%) and refractory epilepsy (100%) in patients with mitochondriopathies, which are categorized as energy metabolism disorders based on SSIEM 2012.

According to a study by Birute et al., extensive search of OMIM, PubMed, and MEDLINE databases showed that the majority of IEM cases (87%) with epilepsy are autosomal recessive, while 6% are mitochondrial, 4% are X-linked, and 3% are autosomal dominant. The prevalence of consanguineous marriage was 74% in our patients, which potentially increases the risk of IEM and highlights the importance of promoting public knowledge and preventive policies.

**In Conclusion,** Neurometabolic disorders are rare, but treatable causes of epilepsy and require timely and accurate diagnosis, besides specific treatment to prevent the irreversible outcomes.
